# Macrophages and platelets in liver fibrosis and hepatocellular carcinoma

**DOI:** 10.3389/fimmu.2023.1277808

**Published:** 2023-12-05

**Authors:** Martina Casari, Dominik Siegl, Carsten Deppermann, Detlef Schuppan

**Affiliations:** ^1^Center for Thrombosis and Hemostasis, University Medical Center of the Johannes Gutenberg-University, Mainz, Germany; ^2^Institute for Translational Immunology, University Medical Center of the Johannes Gutenberg-University, Mainz, Germany; ^3^Research Center for Immune Therapy Forschungszentrum für Immuntherapie (FZI), University Medical Center of the Johannes Gutenberg-University, Mainz, Germany; ^4^Division of Gastroenterology, Beth Israel Deaconess Medical Center, Harvard Medical School, Boston, MA, United States

**Keywords:** angiogenesis, collagen, endothelial cell, Kupffer cell, MMP, monocyte, myofibroblast, MASH

## Abstract

During fibrosis, (myo)fibroblasts deposit large amounts of extracellular matrix proteins, thereby replacing healthy functional tissue. In liver fibrosis, this leads to the loss of hepatocyte function, portal hypertension, variceal bleeding, and increased susceptibility to infection. At an early stage, liver fibrosis is a dynamic and reversible process, however, from the cirrhotic stage, there is significant progression to hepatocellular carcinoma. Both liver-resident macrophages (Kupffer cells) and monocyte-derived macrophages are important drivers of fibrosis progression, but can also induce its regression once triggers of chronic inflammation are eliminated. In liver cancer, they are attracted to the tumor site to become tumor-associated macrophages (TAMs) polarized towards a M2- anti-inflammatory/tumor-promoting phenotype. Besides their role in thrombosis and hemostasis, platelets can also stimulate fibrosis and tumor development by secreting profibrogenic factors and regulating the innate immune response, e.g., by interacting with monocytes and macrophages. Here, we review recent literature on the role of macrophages and platelets and their interplay in liver fibrosis and hepatocellular carcinoma.

## Liver physiology in health and disease

The liver is a highly diversified organ and as such is involved in numerous key metabolic processes e.g., of lipids, proteins, complex carbohydrates, glucose and xenobiotics (1–4). Moreover, the liver plays an important role in immune regulation (5) and hemostasis. Apart from most coagulation factors (6), hepatocytes synthesize thrombopoietin (TPO), the master regulator in platelet production and maintenance (7). While chronic liver diseases leading to advanced fibrosis and cirrhosis are associated with bleeding disorders and thrombocytopenia due to splenomegaly and hepatocyte synthetic failure, hypercoagulability and thrombosis add to the picture, which illustrates the impact that disbalances within the liver can have on the tightly controlled effector cascades in hemostasis (8, 9).

The liver receives blood from the portal vein as well as from the hepatic artery and comes into close contact with nutrients, microbial metabolites, and antigens, which originate from the intestine (10). By default, the liver’s immune milieu has been primed for tolerance during early childhood, usually suppressing immune reactions against gut-derived antigens that are sensed as harmless or beneficial to the body (11–13). Exogenous stimuli can overcome the tolerance promoting role of liver (innate) immune and sinusoidal endothelial cells, leading to chronic liver diseases (CLDs). These CLDs, when left untreated, can progress to fibrosis, cirrhosis and hepatocellular carcinoma (HCC) which account for two million deaths per year and a much higher morbidity (14).

Triggers that can drive CLDs are persistent viral hepatitis B and C, alcohol abuse leading to alcohol-associated liver disease (ALD), autoimmune hepatitis including (autoimmune) biliary diseases, genetic liver diseases or drug-induced liver injury. Moreover, today the most common CLD is metabolic dysfunction-associated steatotic liver disease (MASLD), formerly known as nonalcoholic fatty liver disease, with a global prevalence of about 20-40% (15, 16). The definition was recently amended to include at least one of four cardiometabolic risk factors associated with steatohepatitis, namely obesity, type 2 diabetes, hypertension and dyslipidemia (triglycerides/cholesterol) (17). Furthermore, additional ‘second hits’ determine the severity of MASLD, including an association with increased alcohol consumption, now defined as MetALD (16). MASLD can be further differentiated into metabolic dysfunction-associated steatohepatitis (MASH), formerly non-alcoholic steatohepatitis, characterized by chronic inflammation, including hepatocyte damage (lipoapoptosis and ballooning), that promote progressive liver fibrosis (18). MASH is found in up to 20% of MASLD patients and incurs a high risk of cirrhosis development, where 9-25% of the patients show a cirrhotic liver within 5-10 years (19). Importantly, in CLD with underlying cirrhosis vs. its absence, the risk for developing HCC is increased up to 200-fold, with an incidence of 1-6% once cirrhosis has developed (20). Here, we will give a short overview about the pathomechanisms of liver fibrosis as they relate to the role of macrophages and platelets, and especially their interactions in liver fibrosis. While this research has just begun, it promises to not only yield novel insights into the pathogenesis of fibrosis progression but also reveal new drivers of HCC development that may lead to advanced antifibrotic or HCC-directed therapies.

## Pathophysiology of liver fibrosis

Fibrosis defines a pathological wound healing response, and fibrosis progression results from ‘wounds that do not heal’ (18, 21–24). Here, activated (myo)fibroblasts express and deposit excessive amounts of extracellular matrix (ECM) proteins, most prominently interstitial collagens type I, III and VI, and basement membrane collagen type IV, but also hundreds of other collagenous and non-collagenous proteins, glycosaminoglycans and proteoglycans. This excess ECM finally replaces healthy, functionally important cells and changes the tissue’s vascular architecture, in the body’s attempt to maintain organ integrity at the expense of function (25–27). In the liver, this leads to progressive loss of hepatocyte function, prehepatic (portal) hypertension with complications like esophageal variceal bleeding, ascites, susceptibility to infection and hepatic encephalopathy due to loss of detoxification of general and intestinal (microbial) metabolites (28, 29). The ECM composition is altered in active fibrosis and the process itself is highly dynamic, showing both upregulated formation (fibrogenesis) and degradation (fibrolysis) of ECM components, usually in favor of fibrogenesis (30). In general, the ECM is a scaffold to which cells bind to and interact with each other. It also directs cellular signaling, polarization and differentiation by engaging specific ECM receptors and by binding cytokines or hormones that are released from these ECM stores into the circulation upon ECM remodeling, leading to the concept of defining the ECM as an ‘endocrine organ’ (31).

**Figure 1:**
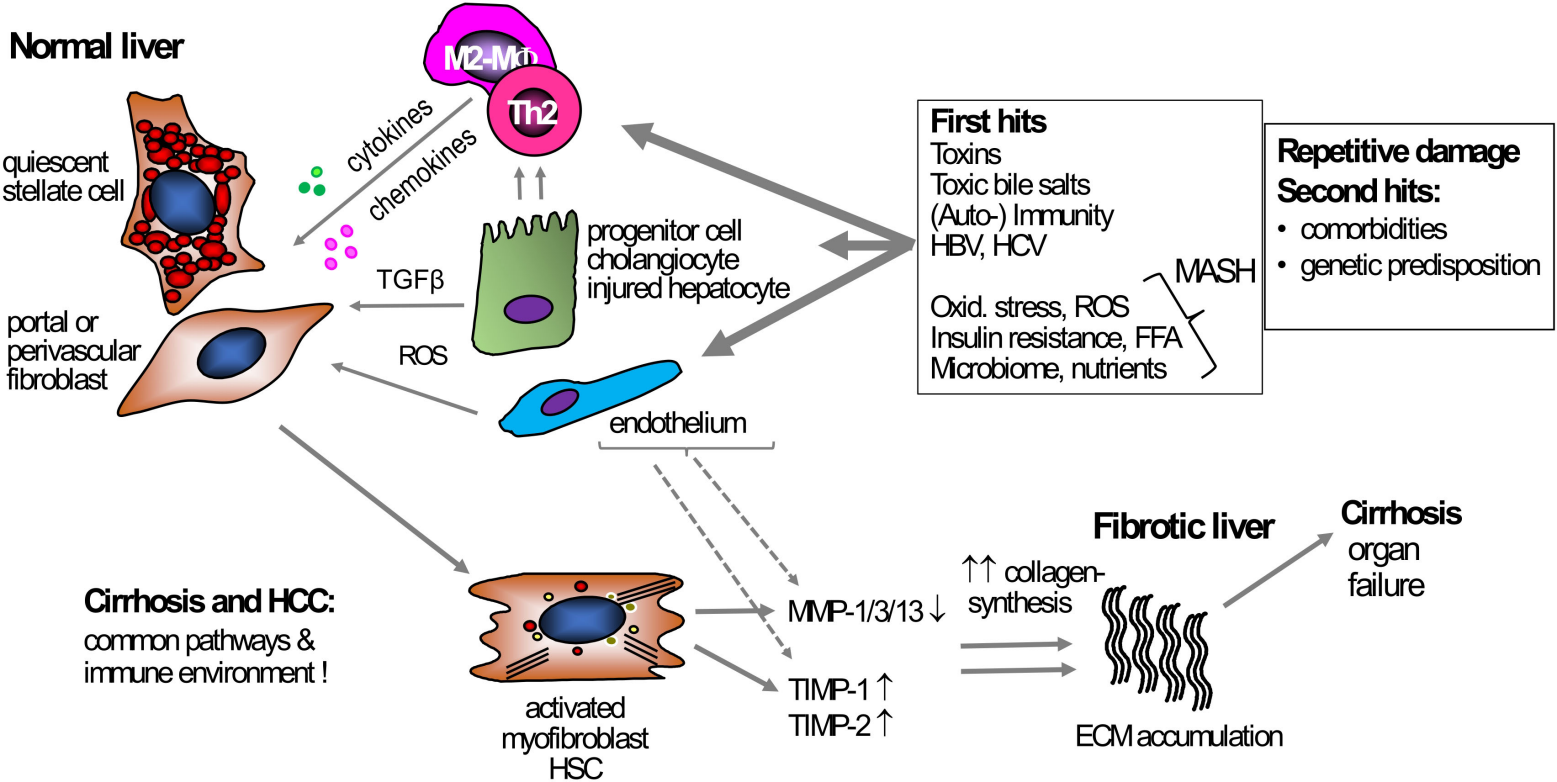
Mechanism of liver fibrosis. Under normal conditions, HSCs and portal or perivascular fibroblasts, the primary effector cells, are in a quiescent state and support steady-state ECM production. Various triggers can act as primary causes inducing chronic liver damage, e.g., exposure to toxins, chronic hepatitis B- or C infection, or metabolic and oxidative stress in MASH. These triggers induce hepatocyte damage that starts a pro-inflammatory response, usually initiated by monocytes and macrophages, but also T cells. Besides these primary hits triggering inflammation, secondary hits like unhealthy nutrition, microbiota, or genetic predispositions can contribute to, enhance, and prolong the fibrogenic response. During inflammation, TGFβ, secreted by, e.g., macrophages and damaged hepatocytes, induces HSC and (portal) fibroblast activation, leading to increased proliferation, migration, and subsequent excessive ECM production and deposition, resulting in fibrosis and (vascular) architectural remodeling. Fibrogenesis is usually accompanied by suppressed fibrolysis, exemplified by an increased expression of TIMP-1 and -2 that inhibit ECM removal by blocking MMP function. Several primary and secondary hits that are driving chronic liver inflammation can be addressed causally, for example via potent antiviral therapy for hepatitis B or C, lifestyle intervention for MASH, or abstinence for alcohol-associated liver disease. However, once these diseases have progressed to cirrhosis, direct antifibrotic therapies are needed to induce fibrosis regression. ECM, extracellular matrix; HSC, hepatic stellate cell; MF, (myo)fibroblast; MMP, matrix metalloproteinases; MASH, metabolic dysfunction-associated steatohepatitis ROS, reactive oxygen species; TGFβ, transforming growth factor beta; TIMP, tissue inhibitor of metalloproteinases; TLR4, toll-like receptor 4.

Interestingly, activated (myo)fibroblasts, the major cellular producers of excessive ECM and thus scar tissue, are induced in the liver and other organs during inflammation, and expand when inflammation becomes chronic (**Figure 1**) (32). The dominant source of (myo)fibroblasts varies, dependent on the etiology and pathophysiology of fibrosis and, e.g., murine models employed. While for the murine model induced by the hepatotoxin CCl_4_, activated hepatic stellate cells (HSC), which serve as sinusoidal pericytes residing in the hepatic parenchyma, become the main ECM-producing cells, portal fibroblasts are the dominant ECM producer in cholestatic fibrosis models (33–35). In both fibrosis scenarios, these two cell types are the source of >90% of all (myo)fibroblasts, while there is only a minor contribution of fibrocytes, cells likely originating from circulating monocytes that are recruited to injured organs (36). These findings are relevant when developing antifibrotic therapies since the cellular origin of the activated (myo)fibroblasts can have an impact on the treatment response. As an example, pharmacological stimulation of soluble guanylate cyclase or inhibition of fibroblast activation protein is effective in CCl_4_-induced liver fibrosis dominated by activated HSCs, but ineffective in the bile duct ligation model, dominated by activated portal (myo)fibroblasts (33, 37), while the opposite was observed when liver fibrotic mice were treated with an antagonist to the endothelin A receptor, an integrin αvβ6 antagonist, or a TGFβ2 inhibiting antisense oligonucleotide (38–40).

Irrespective of the prominent fibrogenic cell type, inflammation is usually necessary for fibrosis initiation. An example is lipotoxicity in hepatocytes, a hallmark of MASH. Lipid overloading and especially the inability of the hepatocytes to handle the excess lipids by safe storing in lipid droplets or to safely degrade the excess lipid via, e.g., the mitochondria or peroxysomes, enhances mitochondrial and hepatocellular oxidative stress and dysfunction (41) which is linked to endoplasmatic reticulum (ER) stress induced via the unfolded protein response (42). Impaired autophagy, increased mitophagy and accumulation of toxic oxidized lipids, including epoxides, glycerophospholipids and sphingolipids further promote hepatocyte injury and apoptosis (43–46). The thus injured and necroapoptotic hepatocytes secrete danger signals like damage-associated molecular patterns (DAMPS) as high mobility group protein 1 (HMBG1) (47), mitochondrial remnants (48), or exosomes that contain immune regulatory micro RNAs and chemokines like CCL2 and CXCL1 (46). These metabolites and signaling molecules can directly activate HSCs or portal fibroblasts, but also activate and attract immune cells, especially macrophages/Kupffer cells (49) that further increase local inflammation and shape the fibrotic response. During steady state, liver sinusoidal endothelial cells (LSECs) are highly fenestrated and endocytotically active. They control and induce quiescence of the adjacent HSCs that serve as sinusoidal pericytes (50, 51). With a disrupted intestinal barrier, LSECs encounter an increased amount of gut-derived pathogen-associated molecular patterns (PAMPS), which leads to their transformation, involving, e.g., heat shock protein (Hsp) 90 acetylation and subsequent reduction of homeostatic nitric oxide production (52). When this occurs, LSECs promote sinusoidal capillarization, express inflammatory cytokines like TNF-α, CCL2, and CCL5, thereby recruiting inflammatory immune cells, further stimulating (myo)fibroblast transactivation, thus losing their usual ability to control HSC activation (51, 53).

These select examples show how chronic inflammation in the liver, triggered by viral, metabolic, toxic and intestine-derived stimuli can initiate a vicious cycle creating a continuing wounding response, therefore tilting the tight balance of pro-and antifibrotic mechanisms that occur in acute wound healing towards a constant activation of (myo)fibroblasts, with excess ECM deposition and finally liver cirrhosis and failure (18, 28, 54).

## Pathophysiology of liver cancer

Globally, primary liver cancer (hepatocellular carcinoma, HCC, 75-85%; cholangiocarcinoma, CCC, 10-15%, some rare entities like fibrolamellar carcinoma) is the third leading cause of cancer-related death and the sixth most commonly diagnosed cancer (55). 70-90% of all primary liver cancers develop in the context of CLD and cirrhosis (56). Most CLD patients show no or few clinical symptoms or anomalies in the pre-cirrhotic stage, resulting in late-stage diagnosis and poor prognosis, exemplified in a population-based cohort study, where 75% of the patients had no or minor complications of cirrhosis at entry (57). In this context, several population-based studies assessed a prevalence of significant fibrosis, i.e., stage 2-4 as determined by biopsy, in 1.8-12.6% of the general population, the range being explained mainly by the prevalence of viral hepatitis, the exposure to aflatoxin, MASH or alcohol abuse (55, 58), and as being related to the quality of the health care system (59–62). This illustrates the need for earlier diagnosis and effective therapies to prevent progression to cirrhosis and HCC. Since advanced fibrosis is the major risk factor for HCC development, the risk factors that promote fibrosis are also important cofactors for HCC development.

In MASH, the hypercaloric diet promotes hepatocyte oxidative stress. The resulting H_2_O_2_ and ROS production can directly activate HSCs, transform latent ECM-bound TGFβ1 into its biologically active form, thereby driving their transformation into fibrogenic (myo)fibroblasts (63–66). H_2_O_2_ also acts as proinflammatory molecule leading to Kupffer cell activation, inducing an inflammatory response that further drives fibrosis, which can result in a closed loop of chronic inflammation, hepatocyte necro-apoptosis (lipo-apoptosis), further enhancing ROS-production and fibrogenesis (49, 63, 64).

Moreover, apart from chronic inflammation that links fibrosis and HCC, the altered ECM in advanced fibrosis itself can facilitate further fibrosis progression, HCC/CCC evolution, and metastasis. The ECM determines the immune environment in cancer to serve as substrate to which immune cells, especially dendritic cells, macrophages and T cells bind and by which they are functionally modulated through, e.g., sensing ECM stiffness via integrin-receptor mediated ECM signals (67–71). Also, HCC shows cancer-specific ECM remodeling with distinct disease-related ECM signatures that exhibit prognostic value (72, 73). Moreover, increased ECM stiffness can induce exosome secretion by tumor cells that was shown to promote cancer growth via paracrine Notch signaling, remodeling of the tumor microenvironment (74) as well as the activation of Yes-associated protein (YAP) and the YAP/TEA domain transcription factor 4 (TEAD4) complex in cancer cells (75, 76).

## The role of mesenchymal cells in the HCC/CCC microenvironment

Detailed mechanisms and drivers of HCC and CCC in the non-fibrotic and especially in the fibrotic liver are major current research areas, with a clear view towards clinical translation (77, 78). As in other cancers, the malignant transformation of hepatocytes, bile duct epithelia and hepatic progenitor cells is a multifactorial and multistep process, driven by complex and deregulated signaling pathways and cell-cell interactions, involving the tumor microenvironment (TME). The TME includes LSECs, cancer associated fibroblasts (CAFs) that are related to activated HSCs and (myo)fibroblasts (68, 79), and especially immune cells, mainly myeloid and T cell subsets (80). Recent examples highlighting the important role of non-immune cells in modulating HCC/CCC growth and dedifferentiation are findings that e.g., quiescent HSC-derived hepatocyte growth factor promotes epithelial cancer growth (81), or that Musashi RNA binding protein 2 (MSI2) downstream signaling in (myo)fibroblasts leads to IL-6 and IL-11 secretion, cytokines that stimulate cancer cell proliferation (82). In addition, activated HSCs secrete extracellular vesicles containing hexokinase 1 that are engulfed by neighboring HCC cells, leading to accelerated glycolysis and the promotion of HCC progression (83). In LSECs, simvastatin-loaded nanoparticles alleviated sinusoidal capillarization, restored quiescence of activated HSCs by stimulation of Krüppel-like factor 2/NO signaling in LSECs, and upregulated CXCL16 expression resulting in the recruitment of natural killer T cells (NKT), which suppressed HCC progression (84). These few examples illustrate how a disrupted tissue homeostasis induces a tumor-promoting TME not only by directly modifying the immune cell environment, but also by altering the non-immune cell TME, mainly represented by CAFs (HSCs/(myo)fibroblasts) and LSECs.

## Macrophage subsets in the liver

Macrophages are innate immune cells, present in every organ of the body (85) and are the most abundant immune cell population in the liver (86). They ensure tissue integrity by phagocytosis of cellular debris, waste products and apoptotic cells (87–89), and act as first line defense against pathogens. Macrophages express various pattern recognition receptors (PRRs) like Toll-like receptors (TLRs) or NOD-like receptors (NLRs). Their activation by PAMPs leads to activations of nuclear factor-κB (NF-κB), interferon regulatory factors (IRFs) and mitogen-activated protein kinase (MAPKs) and the expression of downstream effector cytokines and chemokines, orchestrating an inflammatory response (90, 91).

Within the liver, two different macrophage subsets of different origin can be distinguished. First, Kupffer cells are tissue resident macrophages with self-renewing capacity, originating from the yolk sack (92). They sense gut-derived antigens, which the liver is constantly exposed to, and play a major role in maintaining tissue homeostasis by inducing tolerance to the many (harmless) nutrient- or microbial-derived antigens that pass through the liver immediately after intestinal digestion and resorption, for example via secretion of IL-10 and by favoring the expansion of tolerogenic T regulatory cells (Treg) (93). Second, during infection or in situations when the natural default tolerance of the liver is overrun, monocyte-derived macrophages (MoM) are recruited to the site of inflammation, where they trigger an initially protective inflammatory response, followed by their differentiation into pro-inflammatory macrophages.

**Figure 2:**
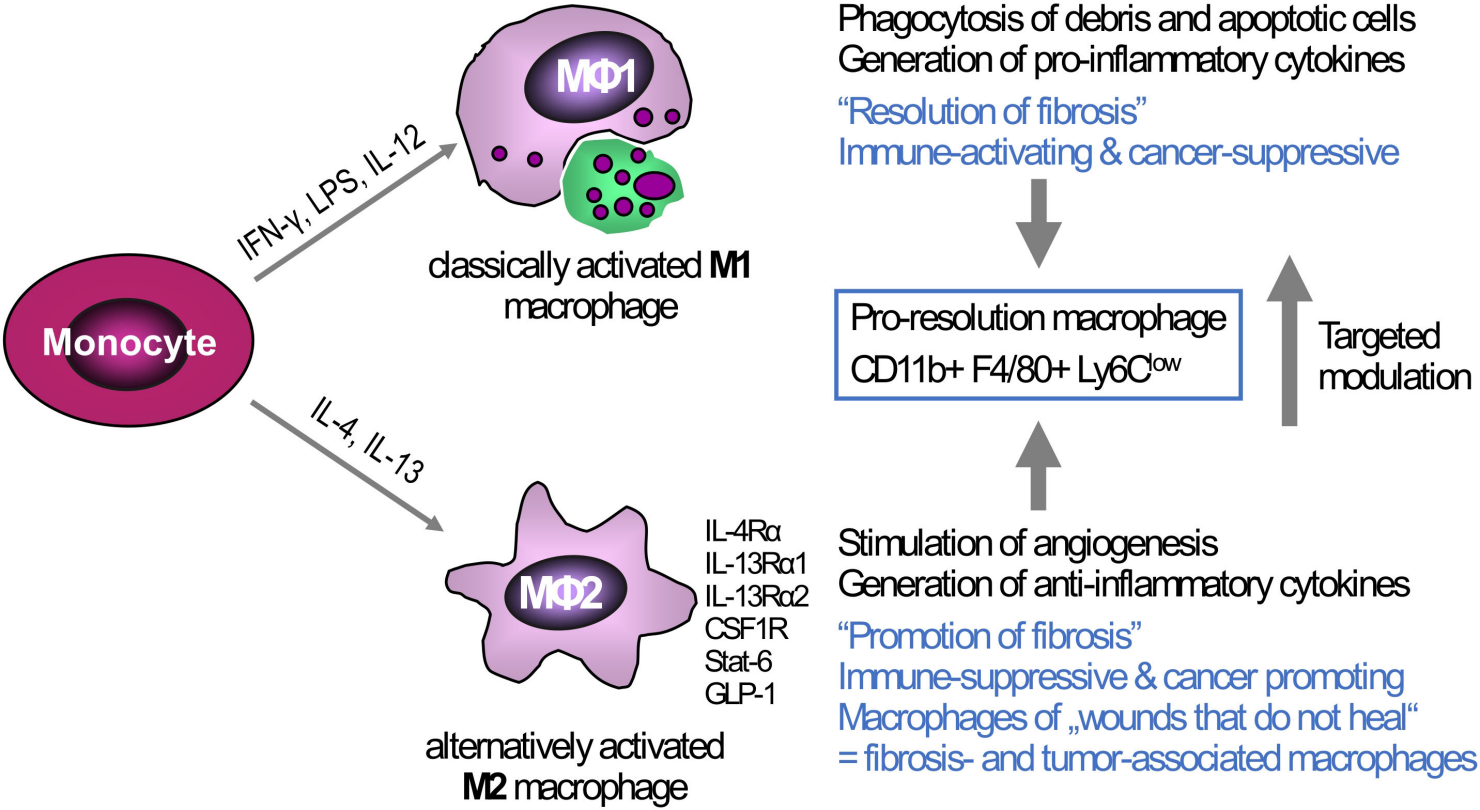
Triggers of macrophage polarization and the resulting phenotypes: Tissue-infiltrating monocytes as well as tissue-resident Kupffer cells are the sources of liver macrophages. *In vitro*, monocytes/macrophages can be polarized towards a M1-type (classically activated macrophages) via IFN-γ, LPS, or IL-12, or towards M2-type via IL-4 and IL-13 (alternatively activated macrophages). The M1-type is rather associated with high(er) phagocytotic activity and the production of pro-inflammatory cytokines that can induce ECM breakdown and a prominent anti-cancer response, This M1 phenotype can switch to a low or anti-inflammatory M2-type that suppresses inflammation but at the same time promotes fibrogenesis, e.g., by release of TGFβ1, and cancer growth by generating a tolerogenic cancer microenvironment. While the major *in vitro* phenotypes only exemplify the extremes of macrophage polarization, *in vivo* macrophages show high plasticity and therefore can exhibit both M1-type and M2-type characteristics at the same time. Thus, in liver fibrosis of different etiologies both M1-and M2-type macrophages can induce and shape liver inflammation, while a subset that is defined as “pro-resolution macrophages” shows both M1- and M2-type characteristics, acting both anti-inflammatory and fibrolytic, as also shown by their transcriptomic profiles. One therapeutic strategy, already showing promise in preclinical studies is the targeted modulation of macrophage functional phenotypes to overcome liver fibrosis and/or cancer. Examples of possible phenotype ‘switches’ are CSFR1, macrophage colony- stimulating factor receptor 1; GLP-1, glucagon-like peptide 1; IFN-γ, interferon-γ; IL-4RA, interleukin-4 receptor α; IL-13RA, interleukin-13 receptor α; LPS, lipopolysaccharide; LY6C, lymphocyte antigen 6C; STAT6, signal transducer and activator of transcription 6.

In general, MoM (and to a lesser degree Kupffer cells) show high plasticity. Mills et al. coined the term ‘M1 vs. M2 macrophage polarization’ based on their findings that macrophages of C57BL/6 mice (Th1 T cell predominant, classically activated, pro-inflammatory M1-type macrophages) were more easily stimulated to produce NO in comparison to Th2 T cell predominant mouse strains (BALB/c, alternatively activated M2-type macrophages) (94). *In vitro*, the M1 phenotype is induced via LPS and IFN-γ resulting in pro-inflammatory activity including pathogen clearing. *In vitro*, the M2 phenotype is induced by IL-4 and IL-13 and was initially characterized as anti-inflammatory, playing a prominent role in tissue repair (95) (**Figure 2**). However, the picture is more complex, with e.g., at least four M2-subtypes, some of them with pro-inflammatory characteristics (96, 97). Newer techniques, especially single-cell RNA sequencing, identified even more different Kupffer cell and MoM populations in mice and humans (98–100). A distinct subpopulation defined as scar-associated TREM2+ CD9+ macrophages was described, originating from MoM, that acts pro-fibrotic by promoting HSC collagen production and proliferation (101). Others described TREM2+ macrophages as lipid-associated macrophages (102, 103) that were shown to be less responsive to TLR4 signaling then Kupffer cells (104). Fabre and colleagues went one step further in characterizing the scar-associated macrophages in pulmonary and hepatic fibrosis of both mice and men using single-cell RNA datasets to identify a subpopulation of macrophages that, in addition to TREM2 and CD9, expressed osteopontin (SPP1), osteoactivin (GPNMB), fatty acid binding protein 5 (FABP5) and CD63. Interestingly, this subpopulation was found to be enriched at scarring sites (105). Therefore, major efforts are currently directed to better define profibrotic vs. fibrolytic (pro-resolution) liver macrophages and specific subpopulations to identify novel therapeutic targets and strategies for antifibrotic treatment (24, 49, 106–109).

## The role of macrophages in fibrosis initiation, progression, and resolution

Pleiotropic effects of macrophages in fibrosis initiation, progression or resolution have been described. Macrophage depletion in the CCl_4_ model of progressive parenchymal liver fibrosis led to a decrease of activated (myo)fibroblasts and attenuated collagen accumulation, while depletion after discontinuation of CCl_4_ prevented the otherwise spontaneous fibrosis resolution (110). Later ‘pro-resolution’ macrophages with an expression profile of both M1- and M2-type macrophages were implicated in fibrosis regression (111) (**Figure 2**). Finally, the study of several knockout mice for M2-type macrophage and Th2 cell signaling as well as the use of therapeutic IL-4Ra antisense oligonucleotides confirmed that even M2-type macrophage signaling can be pro-fibrotic during active liver inflammation, whereas it can promote fibrolysis during spontaneous fibrosis regression after cessation of the inflammatory stimulus (106).

During acute inflammation, activated Kupffer cells and MoM express pro-inflammatory cytokines like IL-1β, TNFα, and IL-6, but also chemokines like CCL2, CXCL1-3 (112–114), leading to further recruitment of MoM and neutrophils, which enhances the initial inflammatory response. Activated macrophages, especially in later stages of inflammation, as well as activated platelets secrete (active) TGFβ1 in response to injury (115), which is a key fibrogenic cytokine driving fibrogenic HSC and (myo)fibroblast activation (116, 117) via the Smad2-4 transcription factor to enhance ECM production (118). In addition, platelet-derived growth factor (PDGF-BB), which is mainly if not exclusively secreted by platelets, strongly stimulates HSC and (myo)fibroblast proliferation, further promoting the fibrogenic response (119, 120). The importance of recruitment of MoM, orchestrated by CCL2 but also other chemokines, partly derived from neutrophils and other myeloid cells or even activated HSCs, to pave the way for progressive fibrotic disease was recently shown, since early anti-CCR2 siRNA treatment ameliorated parenchymal, CCl_4_-induced liver fibrosis (121).

Many different endogenous and exogenous stimuli can trigger the proinflammatory M1 phenotype. For example, complement factor C5a stimulates pro-inflammatory pathways via C5aR1 on macrophages, and C5aR1ko knockout mice showed a M1- to M2-type macrophage transition and reduced fibrosis in a MASH mouse model (122). HMBG1 secretion from injured hepatocytes induced NLRP3 inflammasome activation in macrophages (123), and fibrinogen-like protein 2 (Fgl2), which was upregulated in liver tissues of cirrhotic patients with underlying hepatitis C infection, promoted M1 polarization (124). Furthermore, autophagy triggered a M2-type, whereas LPS stimulation favored a M1-type macrophage polarization and blocked autophagy (125). Painting the same picture, deficient chaperone-mediated autophagy in macrophages was shown to intensify inflammation in MASH (126). Notably, while the shift from the classical proinflammatory M1-type to M2-type macrophages in chronic inflammation attenuated inflammation, it promoted the fibrotic response in MASH (127). In MASH as in other CLDs, the fluctuating course of periods of acute inflammation followed by a M2-type reparative response may underly fibrosis progression in ‘wounds that do not heal’ (18, 128).

Major effectors of both fibrogenesis and fibrolysis are macrophage-derived matrix metalloproteinases like MMP-9, MMP-12 (111) and MMP-13 (129) that lead to collagen degradation that can either pave the way for architectural tissue remodeling towards fibrosis (130), or lead to collagen degradation and the induction of (myo)fibroblast apoptosis (131). Fibrosis resolution is often induced if the underlying major trigger of chronic (M2-type) inflammation is removed but is usually slow or inefficient in advanced human fibrosis and cirrhosis. Moreover, if the underlying trigger continues, the ongoing remodeling of the ECM, induced by secreted MMPs (132) or proteases like cathepsin S (133), can lead to an excessive secondary accumulation and altered composition of the ECM (134). This contributes not only to fibrosis progression but also to cancer initiation, progression and metastasis, including integrin-mediated stress signaling (15, 21, 67, 128, 135–137).

## The role of tumor-associated macrophages in HCC

Tumor-associated macrophages (TAMs) are major cell types infiltrating most TMEs, accounting for 20-40% of immune cells in HCC (138). They act as important drivers of cancer initiation and progression (139). In the liver, tissue-resident Kupffer cells as well as MoM can differentiate to TAMs (140–142), and especially MoM are chemoattracted to the tumor site via the CCR2-CCL2 axis (143). Within the TME, TAMs are turned to a M2 anti-inflammatory and tumor-promoting phenotype by cancer cells in various ways. For example, HCC cells secrete exosomes that contain miRNA-21-5p, which induces M2-type polarization (144), or they overexpress the transferrin receptor, necessary for ferrous iron uptake, and the resulting lower iron concentration in TAMs favors their M2-type polarization (145). Furthermore, metabolic byproducts of cancer cells like lactic acid or succinate drive the TAM phenotype via induction of hypoxia-inducible factor 1a (HIF1a) signaling, that increases TAM expression of e.g., vascular endothelial growth factor (VEGF), arginase 1, found in inflammatory zone (Fizz1) and macrophage galactose-type lectin-1 (Gal-1) (146, 147).

The expression profile and mediator secretion of TAMs is highly immunosuppressive and strongly supports the outgrowth of pre-neoplastic lesions, tumor development and metastasis, mainly by inhibition of cytotoxic CD8+ T cell responses directed to the cancer cells (148, 149).

Thus, TAMs secrete cytokines like IL-8 or IL-10 that stimulate tumor proliferation (150–152), Gal-1 that activates the pro-cancerous mTor-Akt pathway and induces limited autophagy in cancer cells that both promote HCC growth (153). TAMs and the cancer cells are the major producers of VEGF that triggers neo-angiogenesis, supporting the tumor’s nutrient supply (154) and facilitating metastasis. TAMs upregulate carbonic anhydrase XII expression, which secures their survival in the acidic microenvironment but also triggers production of CCL8, VEGFA and MMP9, further supporting neo-angiogenesis, epithelial-mesenchymal transition and metastasis of cancer cells (155, 156).

Of interest, TAMs also interact with cancer-associated fibroblasts (CAFs). CAFs are characterized as activated (myo)fibroblasts, another often abundant, heterogeneous class of cells in the TME. Single-cell RNA techniques could unravel that the interaction of TAMs and CAFs leads to ECM remodeling and the generation of a desmoplastic shell, which hinders lymphocytes to infiltrate the tumor cores (157). The interaction was also found in single-cell RNA datasets in HCC patients, where osteopontin, produced by TAMs, bound to latent TGFβ1 produced by CAFs, illustrating the close interaction of both cell types that potentially can lead to TME remodeling (158). TAM-secreted osteopontin can also directly impede CD8+ cytotoxic T cell function via CD44 signaling on T cells, promoting T cell exhaustion phenotypes (159). A recent study could show that osteopontin (encoded by the SPP1 gene) expression of TAMs indeed holds prognostic value. The authors analyzed human cancer single-cell RNA datasets, revealing that the ratio of CXCL9:SPP1 mirrors the properties of immune cell infiltration and an anti-tumor immune response in many solid cancer types. Of note, the CXCL9:SPP1 ratio was not overlapping with classical M1- and M2-type markers (160). Finally, programmed death ligand 1 (PD-L1) was found to be mostly expressed on TAMs in the TME, suppressing T cell activity (161, 162) and indoleamine 2, 3-dioxygenase (IDO) expressing TAMs suppressed T cell expansion, while supporting Treg proliferation (163).

One highly interesting example of TAM modulation that already entered clinical trials targets Clever-1 (common lymphatic endothelial and vascular endothelial receptor-1), which is prominently expressed on monocytes and macrophages. Preclinical studies showed that human monocytes expressing high levels of Clever-1 impaired Th1 T cell activation, which was reversed via siRNA knockdown or a blocking antibody (164) and that targeting Clever-1 in TAMs via macrophage-specific genetic knockout or via antibody blockade retarded the growth of LLC1 Lewis lung carcinoma cells *in vivo*, by inducing a robust CD8 T cell response (165). A phase II clinical trial testing Clever-1 inhibition using a humanized anti-Clever-1 antibody in 10 distinct, advanced solid tumor types (e.g., melanoma, pancreatic, liver cancer) already showed promising results (166, 167). Finally, Clever-1 on TAMs was recently shown to be responsible for epidermal growth factor (EGF) clearance, a highly relevant tumor promoter (168).

## Physiologic role of platelets

Platelets are small anucleate cell fragments (2-4 µm diameter in humans) that, together with red blood cells, represent the most abundant cells in circulation. The role of platelets was described for the first time in the 19th century by Bizzozero, who observed that platelets were the component of the blood to adhere to damaged blood vessel walls *in vivo* and *in vitro* (169, 170). Platelets have an average life span of 8-10 days in humans and approximately 5 days in mice (171, 172). Thus, platelet turnover is high and their production (thrombopoiesis) by bone marrow megakaryocytes (MKs) is a strictly regulated process (173). Megakaryocytes differentiate from hematopoietic stem cells, and once mature, extend dynamic protrusions, called proplatelets, into bone marrow sinusoids which are then further fragmented to platelets by the shear forces present in vessels (174, 175).

Once released into the bloodstream, platelets primarily function as regulators of hemostasis, circulating and continuously scanning the vascular environment. Platelet activation and thrombus formation occur at sites of vessel injury in a coordinated process that involves tethering, rolling, activation, and firm adhesion. Following endothelial damage, thrombogenic subendothelial ECM proteins like collagen and von Willebrand factor (VWF) get exposed to the blood. VWF binds to collagen fibers and captures platelets from the circulation through the platelet receptor complex glycoprotein (GP) Ib/IX/V (176). This interaction with immobilized VWF enables platelets to bind to the exposed collagen via the GPVI receptor (177). GPVI is associated with the Fc receptor (FcR) γ-chain, which bears an immunoreceptor tyrosine-based activation motif (ITAM) for signal transduction enabling platelet activation (178). These first steps of platelet activation trigger downstream signaling pathways which lead to increased cytosolic Ca^2+^ levels, cytoskeletal rearrangements, degranulation, and integrin activation. Three types of granules can be distinguished within platelets: α-granules, dense or δ- granules, and lysosomes (179, 180). The release of α- and dense granule content enriches the local environment with a multitude of bioactive molecules. Dense granules contain mainly non-protein compounds including calcium, ATP, ADP, serotonin (5-HT), and epinephrine, which can activate platelets in an autocrine way through surface receptors to further strengthen platelet activation (181). On the other hand, α-granules contain more than 300 different proteins involved in coagulation, platelet adhesion, inflammation, wound healing, and angiogenesis (182). Finally, platelet activation shifts several β_1_ and β_3_ integrins to their high-affinity, ligand-binding state, among them integrin α_IIb_β_3_ (GPIIb/IIIa). Activated α_IIb_β_3_ binds to fibrinogen, supporting platelet-platelet aggregation and adhesion to subendothelial ECM proteins (183), but also enables binding to other soluble plasma proteins, including VWF and fibronectin, thereby facilitating stable platelet aggregation and thrombus formation (184).

## The role of platelets beyond hemostasis

In the previous paragraph, we introduced the role of platelets in hemostasis, however, these small anucleate cells are also involved in other pathophysiological processes. Platelets have been observed to play a role in angiogenesis, inflammation, bacterial and viral infection, cancer, tissue regeneration, and fibrosis (185). Platelets can interact with and stimulate cells of the innate and adaptive immune system, mainly monocytes/macrophages, neutrophils, and lymphocytes, thus shaping the immune response.

## Platelet-monocyte/macrophage interaction

**Figure 3:**
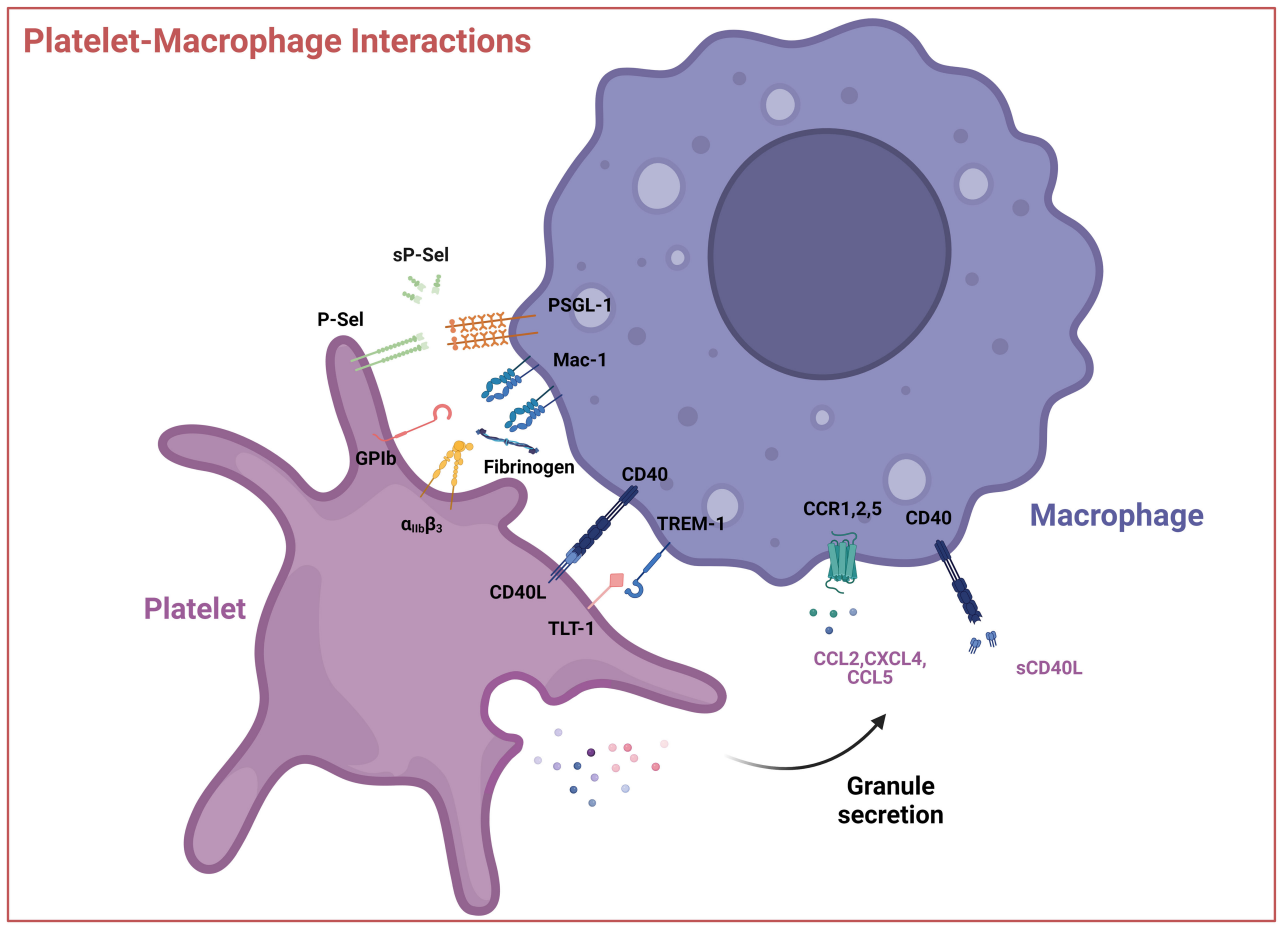
Interactions between platelets and macrophages. Interactions occur via direct contact between platelet cell surface receptors GPIb, P-Selectin, integrin α_IIb_β_3_, CD40L, and TLT-1 with macrophage receptors like Mac-1 or TREM-1 or through soluble mediators like CCL2 (MCP-1), CXCL4 (PF4), CCL5 (RANTES) and sCD40L. These interactions can result in the activation of the platelet, macrophage, or both. P-sel – P-selectin, sP-sel – soluble P-selectin, PSGL-1 – P-selectin glycoprotein ligand, GPIb – glycoprotein Ib, Mac-1 – integrin α_M_β_2_, α_IIb_β_3_, – Integrin α_IIb_β_3_, TREM-1 – Triggering receptor expressed on myeloid cells 1, TLT-1 - TREM-like transcript 1, CCL2 – CC-chemokine ligand 2, CXCL4 – (CXC motif) ligand 4, CCL5 – CC-chemokine ligand 5, sCD40L – soluble CD40 ligand. Created with BioRender.com.

Monocytes and macrophages are key regulators of innate and adaptive immunity. During homeostasis and especially inflammation, monocytes can enter tissues and differentiate into macrophages that, depending on signals from the respective microenvironment, acquire different functional phenotypes. Monocytes and macrophages act as sentinel cells that maintain tissue integrity and eliminate damaged cells and pathogens to restore homeostasis (87–89). In prolonged inflammation or infection, they also promote adaptive immune responses aimed at resolution, but may switch towards an anti-inflammatory, but profibrotic and/or cancer promoting phenotype, as described in a previous chapter (95–97). Activated platelets can recruit and interact with monocytes and macrophages, stimulating mutual activation and the release of cytokines. The major direct interaction between platelets and monocytes/macrophages is achieved through P-selectin (CD62P), which is exposed on the platelet surface following the fusion of the α-granule membrane with the platelet surface membrane upon platelet activation. The interaction of P-selectin with monocyte P-selectin ligand 1 (PSGL-1, CD162) is the first step in platelet–monocyte aggregation (186, 187). This interaction is further strengthened by monocyte membrane-activated complex 1 (Mac-1, integrin α_M_β_2_, CD11b/CD18) which can bind to P-selectin (188), GPIbα (189), and other platelet receptors, including junctional adhesion molecule 3 (JAM-3) (190) and intercellular adhesion molecule 2 (ICAM-2) (191), or bridging proteins such as fibrinogen (bound to the integrin α_IIb_β_3_) (192). Mac-1 interaction with the platelet receptor GPIb occurs through its I domain which is homologous to the VWF A1 domain. During this adhesive process, receptor engagement of PSGL-1 and Mac-1 together with platelet-derived inflammatory compounds induces monocyte activation (193, 194). Platelets can also use their surface receptors CD40L and TREM-like transcript 1 protein (TLT-1) to interact with CD40 (195) and monocyte triggering receptor expressed on myeloid cell 1 (TREM-1) on monocytes (196, 197). Monocytes can also be recruited indirectly by platelets: Monocyte chemotactic protein-1 (MCP-1, CC chemokine ligand 2 [CCL2]) is one of the major chemotactic molecules generated within the vessel wall, interacting with CC chemokine receptor 2 (CCR2) on monocytes and macrophages (198, 199) (**Figure 3**). Activated platelets can also modulate MCP-1 and ICAM-1 expression on endothelial cells via an NF-κB–dependent mechanism (200).

Moreover, platelets release CXC motif chemokine ligand 1 (CXCL1), platelet factor 4 (PF4, CXCL4) and CC-chemokine ligand 5 (CCL5, RANTES) (182, 201). RANTES can increase PF4 binding to the monocyte surface, where it enhances monocyte arrest on endothelial cells (202), predominantly mediated by CCR1, a monocyte receptor for RANTES (203). RANTES can form heterodimers with neutrophil HNP1 (human neutrophil peptide 1, alpha-defensin), stimulating monocyte adhesion through CCR5 (204). Disruption of the HNP1–RANTES interaction attenuated monocyte and macrophage recruitment in a mouse model of myocardial infarction (204). PF4 released from activated platelets induces monocyte phagocytosis and triggers respiratory bursts (205) through phosphoinositol-3-kinase PI3K, spleen tyrosine kinase Syk, and p38 mitogen activated (MAP) kinase activation (206). PF4 also induces extracellular signal kinase 1 and 2 (ERK1/2) phosphorylation, which mediates monocyte survival and differentiation as well as Janus kinase (JNK) signaling, which leads to the production and release of cytokines and chemokines, such as CC-motif ligand 3 and 4 (CCL3 and CCL4) *in vitro* (206). Overall, the outcome of platelet-monocyte/macrophage interactions is highly complex and not yet completely understood, especially since platelets are known to induce opposing effects in macrophages depending on the underlying pathophysiological context and experimental model employed (185, 207) (**Figure 3**).

## Platelets in liver disease

Platelet function is tightly connected with the liver (208): the liver is important for the production of thrombopoietin (TPO) (209), the main growth factor controlling thrombopoiesis and coagulation factors, which are involved in hemostasis (6). The liver also clears aged platelets and liver Kupffer cells have recently been identified as major effector cells in this context (210). Patients with acute or chronic liver diseases frequently present with complex alterations in the hemostatic system (211) including reduced levels of coagulation factors and changes in platelet count (212, 213). Thrombocytopenia correlates with the severity of liver dysfunction, fibrosis, portal hypertension, and splenomegaly (214–216). Some patients with liver disease also display platelet functional defects (217). For this reason, the role of platelets in the progression of liver disease is being analyzed more systematically, and depending on the (patho)physiological context, platelets seem to exert either beneficial or detrimental functions.

## Platelets in liver fibrosis

As previously discussed, in the context of fibrosis, the liver shows a qualitatively abnormal and excessive deposition of scar tissue, dominated by the prominent fibril forming type I and type III collagens but also numerous other ECM molecules, through activated hepatic stellate cells (HSCs) and portal fibroblasts, which progressively impairs the normal liver architecture and functionality (18, 24, 25, 27–29). Notably, platelets can play opposing roles in liver fibrosis as they have both pro- and anti-fibrotic effects.

## The antifibrotic and regenerative role of platelets

Clinical evidence showed that a higher platelet count is associated with less fibrosis and that platelet transfusion can ameliorate liver functionality in chronic liver diseases (218–220). Thrombocytopenic mice developed more severe fibrosis when subjected to liver injury by bile duct ligation (221). ATP and hepatocyte growth factor (HGF) from platelet granules may have antifibrotic effects (221, 222). Experiments *in vitro* revealed that a HSCs-platelet co-culture resulted in platelet activation and HGF release, with subsequent downregulation of type I collagen transcript levels in HSC (221). A beneficial effect of platelets was shown in the carbon tetrachloride (CCl_4_) mouse model of liver fibrosis, where treatment with platelet-rich plasma resulted in an attenuation of liver fibrosis (223, 224). Reduced liver fibrosis and increased liver regeneration were also seen upon administration of a TPO receptor agonist in a mouse model of CCl_4_-induced liver fibrosis (225). Platelet-mediated hepatic regeneration depends on the interaction with sinusoidal endothelial cells, Kupffer cells and hepatocytes (226). *In vitro* studies show that platelets promote endothelial production of interleukin-6 (IL-6) and VEGF, inhibiting apoptosis and stimulating hepatocyte proliferation (226–228). Platelet accumulation in the liver is mainly mediated by direct interaction with Kupffer cells (224, 229). Following this interaction, Kupffer cells produce tumor necrosis factor-α (TNF-α) and IL-6, cytokines critical to liver regeneration (230, 231). Platelets also become activated and move through the sinusoidal endothelium and enter the space of Disse where they directly influence hepatocytes (229). Platelets release hepatocyte growth factor (HGF), VEGF, and insulin-like growth factor-1 stimulating hepatocyte survival and differentiation (231).

## The profibrotic role of platelets

On the other hand, there is evidence for a profibrotic role of activated platelets. Liver fibrosis results in platelet activation and aggregation in the liver tissue, close to the fibrotic areas of patients with progressive HCV and MASH-associated fibrosis (120, 232). After activation, platelets release different mediators which are known key drivers of fibrogenesis. These include platelet-derived growth factors, especially PDGF-AB and –BB, and transforming growth factor β1 (TGFβ1) (120, 233). PDGF-B is a potent mitogen and chemotactic factor for activated HSC and (myo)fibroblast. Yoshida et al. observed that mitogenic PDGF-B, in liver fibrosis, was exclusively produced by activated platelets, and a monoclonal blocking antibody against PDGF-B as well as anti-platelet therapy with low-dose aspirin reduced circulating PDGF-B levels and significantly ameliorated liver fibrosis in two mouse models of advanced biliary fibrosis (120). Accordingly, platelet-specific depletion of TGFβ1 decreased CCl_4_-induced liver fibrosis by reducing profibrotic signaling and collagen synthesis in HSCs (234). Profibrotic effects of platelets were also attributed to VWF (235), serotonin (5-HT), and platelet-derived Sphingosine-1-phosphate (S1P), which activates HSCs to increase collagen secretion and transform into (myo)fibroblasts (236, 237). PF4 could also be involved in the modulation of liver fibrosis, since its genetic deletion in CCl_4_-induced murine liver fibrosis, reduced histological liver damage and fibrosis-related transcript levels, and resulted in the reduction of immune cell infiltration in the liver (232). Additionally, it was reported that PF4, released from platelets, drives the differentiation of a profibrotic macrophage population marked by the expression of *Spp1, Fn1 and Arg1.* Loss of PF4 in mice abolished profibrotic *Spp1*-mediated macrophage differentiation and ameliorated fibrosis after both heart and kidney injury (238). Positive results from the use of antiplatelet therapies were confirmed in two epidemiological studies of liver fibrosis patients with or without aspirin therapy (239, 240). Using different mouse models of MASH, an extensive study showed that antiplatelet therapy (aspirin/clopidogrel, ticagrelor) reduced inflammation and liver fibrosis. The authors demonstrated that liver resident macrophages (Kupffer cells) are important for platelet accumulation in the liver, and that platelet GPIbα appears to be primarily involved in the interaction of platelets with Kupffer cells and the maintenance of MASH. Moreover, Kupffer cell depletion via clodronate liposomes resulted in a significant decrease in intrahepatic platelet numbers, confirming that Kupffer cells recruit platelets to the liver in the setting of MASH (241). Taken together, the role of platelets in liver fibrosis is still not fully understood, since it appears to be dependent on disease etiology and stage, which requires further investigation. The use of different animal models, timing, and conditions could help solve the Janus-faced behavior of platelets observed and help to shed light on their role in fibrosis progression or regression.

## Platelets in cancer and HCC

A relevant role for platelets in cancer was suggested more than 100 years ago when occult carcinomas were identified by the patients’ excessive blood clotting leading to venous thrombosis and embolism (242). Further clinical evidence supported platelets as active players in all steps of tumorigenesis including tumor growth, extravasation, and metastasis (243). Cancer-associated thrombosis is a leading cause of death in cancer patients (244). Accordingly, cancer patients often display elevated platelet counts and/or altered platelet function (245), and thrombocytosis has been associated with an unfavorable prognosis at the time of cancer diagnosis (246, 247). In HCC, thrombocytosis positively correlates with large tumor size, recurrence, and poor response to chemotherapy (248, 249). Increased platelet size (mean platelet volume, MPV), has also been associated with HCC progression (250). Postoperative high platelet-to-lymphocyte ratio (PLR) can predict HCC recurrence and decrease overall survival after surgical liver resection (251). Notably, an elevated platelet count is related to an increased risk of developing extrahepatic metastasis (249), possibly because of platelet-induced tumor cell growth and migration (252). However, for HCC, the relationship between platelet count and tumor development is more complicated, as both thrombocytosis and thrombocytopenia have been described as risk factors for HCC development and poor prognosis (248, 250). This is likely due to the high prevalence of cirrhosis, a key cofactor of HCC evolution that causes splenomegaly and resultant thrombocytopenia (28, 253). Still, in patients with cirrhosis caused by fatty liver disease, a low platelet count was recently included as a reliable marker to predict HCC development (254). In general, thrombocytopenia is used to identify patients with more advanced (cirrhotic) liver disease at risk of developing HCC (255, 256) and to predict mortality of patients with cirrhosis or HCC (257), while thrombocytosis may predict more rapid cancer progression in patients with noncirrhotic HCC (28, 250–253).

## Platelet interaction with cancer cells and the TME

Platelets and tumor cells interact directly or indirectly through the release of soluble mediators. These interactions can result in the alteration of platelet physiology that further supports tumor growth (258). Tumor cells can recruit platelets into hepatic tumor tissue through the release of tumor cell-derived chemokine (CX3C motif) ligand 1 (CX3CL1) (259) and cancer cells can express molecules such as podoplanin and thrombin, which interact with platelet C-type lectin-like receptor 2 (CLEC-2) and protease-activated receptors (PARs) to activate and aggregate platelets (260–263). Cancer cell-derived IgGs activate platelets by binding to platelet FcγRIIa (264). Additionally, soluble factors such as ADP, released by tumor cells, can also activate platelets, probably via P2Y_12_/P2Y_1_ (265). Cancer-induced platelet activation is thought to be one of the reasons why increased thrombosis is observed in cancer patients (244). Activated platelets contribute to cancer growth and metastasis (266).

**Figure 4:**
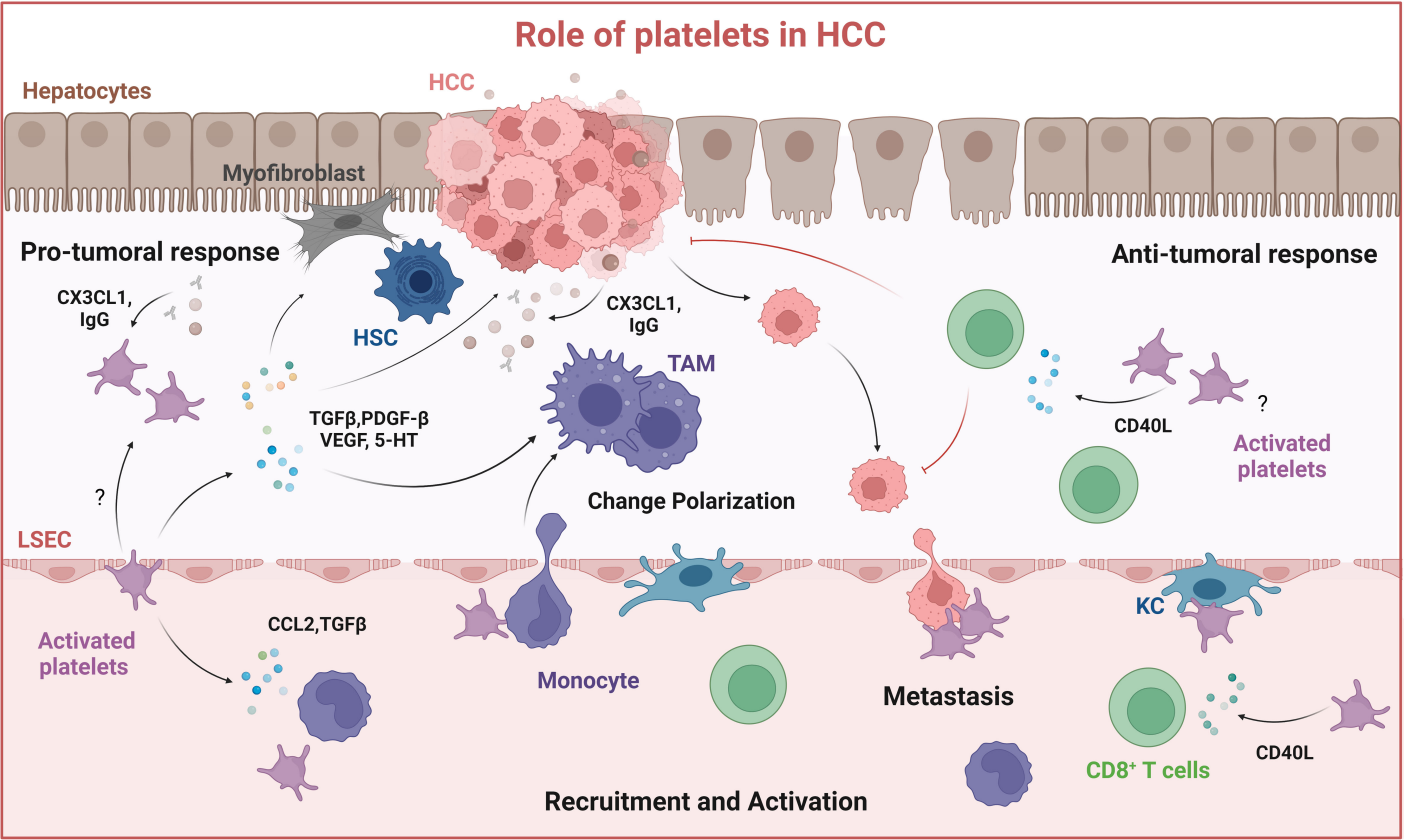
Role of platelets in HCC. Platelets are recruited to the tumor site by interacting with liver resident macrophages (Kupffer cells) and by cancer cells through the release of CX3CL1 and IgG, resulting in platelet activation. Activated platelets release soluble mediators: TGFβ, PDGF-BB, VEGF, and serotonin (5-HT) which contribute to HSC activation, macrophage M2-type polarization into immune suppressive tumor-associated macrophages (TAMs) and thus tumor growth. Platelets can also mediate anti-tumor responses by activating CD8^+^ T cells through releasing CD40L. Created with BioRender.com.

In human HCC biopsies, activated platelets are found close to tumor cells (259, 267, 268) and adhere via their activated α_IIb_β_3_, GPIb-IX-V, and, P-selectin receptors (268, 269). Through these interactions, platelets become activated and secrete factors such as platelet-derived PDGF-BB, TGFβ1, serotonin, and VEGF that support tumor progression and angiogenesis (270–272) (**Figure 4**). Here, platelet TGFβ1 is a general driver of cancer cell epithelial-to-mesenchymal transition (EMT) via activation of the Smad2/3 and NF-kB pathways (273, 274), and of HCC growth both *in vitro* and *in vivo*, where it also suppresses cancer cell Krueppel-like factor 6 (KLF6) expression (275). An *in vitro* study also showed that platelet-derived serotonin could induce the proliferation of three different HCC cell lines (Huh7, HepG2, and Hep3B) (276). In this line, another study reported that intra-platelet serotonin content was correlated to early disease recurrence after liver resection of HCC (277). Besides interacting with cancer cells, platelets recruit leukocytes and interact with LSECs and HSCs (120, 278, 279) affecting the TME. Platelets induce the release of IL-6 from LSECs which enhances hepatocyte proliferation (227). Further, VEGF, which is stored in platelet α-granules increases LSEC fenestration (280). Platelets can also contribute to the formation of an immune suppressive milieu in the TME (243) by secreting chemokines that recruit M2-type macrophages into the TME. As mentioned before, platelets can recruit monocytes into the tissue, for example via CCL2 and its receptor CCR2 on monocytes (198, 199). *In vitro*, human platelet-derived serotonin inhibited TNFα production in stimulated monocytes and macrophages primed for anti-inflammatory signaling (281, 282), and platelets downregulated TNFα production, abrogating the capacity of macrophages to kill tumor cells (283). Platelet-derived microparticles (extracellular vesicles, EVs) also change macrophage polarization. Microparticles generated from platelets contain RANTES, macrophage migration inhibitory factor (MIF), CXCL-12, and IFN-γ that promote the differentiation of monocytes into a M1 macrophage phenotype (284). In contrast, Vasina and colleagues showed that platelet-derived microparticles promoted a macrophage M2-type anti-inflammatory/pro-tumoral phenotype, associated with increased expression of chemokine receptors CCR5 and CXCR4 but not CCR2 (285). Another study showed that platelet EV internalization by primary human macrophages changed the macrophage transcriptome, reduced mRNAs encoding for TNFα, CCL4, and CSF1 while upregulating IL-10, consistent with a M2 phenotype (286). Exosomes originating from platelets can also promote the M2 phenotype by inhibiting the activation of the NLRP3 inflammasome (287). The anti-platelet drug clopidogrel enhanced an anti-tumoral hepatic M1 macrophage phenotype (271, 288). CD40L, TGFβ, and programmed death ligand 1 (PD-L1) are important immune mediators secreted by platelets that interfere with immune cell activation, modulate macrophage polarization, and enable cancer cells to escape from immune destruction (288, 289). These findings suggest that platelets play an important role in mediating the macrophage´s immune response, contributing to their polarization into TAMs in HCC and other solid cancers. However, further research is necessary to fully understand the mechanisms underlying the crosstalk between platelets and TAMs (**Figure 4**).

## Use of antiplatelet therapies to treat HCC

Recently, the use of antiplatelet therapies to treat HCC has gained interest. The administration of aspirin and clopidogrel attenuated development and increased overall survival in a transgenic mouse model of chronic, noncirrhotic, hepatitis B virus (HBV) induced HCC (290). Somewhat paradoxically, this was associated with a reduced intrahepatic accumulation of HBV-specific cytotoxic CD8+ T cells, but explained by attenuated hepatocyte damage by these CD8+ T cells (291). Consistent with the observed antiplatelet effect, clinical studies suggested an association between the use of aspirin and a reduced HCC risk in patients with viral hepatitis (292–294). Platelets are also involved in the promotion of MASH, both in the above-discussed mouse model and correlative human data (241). Additional human studies supported the positive effect of anti-platelet therapy, particularly aspirin, on HCC development in both patients with chronic liver disease and in the general population (292, 295–297). In contrast, a recent study found that platelets inhibited the growth of HCC and liver tumor metastasis in MASLD through the purinergic receptor P2Y12-dependent release of platelet CD40L, which was in part directed through cysteinyl leukotrienes (298) (**Figure 4**). Indeed, blocking the production of cysteinyl leukotrienes using zileuton, partially inhibited the upregulation of plasma CD40L. CD40L leads to CD8+ T cell activation via the CD40 receptor, establishing an anti-tumor response. The authors argued that in their study HCC and MASH were already established, in contrast to other studies that focused on HBV or MASH progression and HCC induction (241, 290, 291, 298). In conclusion, these studies suggest that platelets can contribute to cancer growth and progression in multiple ways, depending, e.g., on their spatiotemporal activation during inflammation, fibrogenesis, and HCC evolution, highlighting the complex roles that platelets can play. Future well-designed studies are needed to further investigate the mechanisms involved in platelet–cancer cell–macrophage interactions.

## Conclusion and outlook

Over the past decades, chronic liver diseases have risen to one of the leading causes of morbidity and mortality worldwide. Recent research has generated increasing evidence that hepatic macrophages and platelets play a key role in liver homeostasis and that their dysregulation promotes chronic liver diseases, by modulating inflammation and driving fibrosis or cancer progression. Novel approaches are being developed to target hepatic macrophages, most of them focusing on four different strategies: 1) reducing the activation of MoMs and Kupffer cells, 2) preventing the influx of MoMs into the liver, 3) reprogramming the macrophage phenotype towards an antifibrotic/pro-resolution phenotype, and 4) inducing a pro-inflammatory and anti-tumoral M1-type macrophage. Recent studies unraveled a profound heterogeneity in the hepatic macrophage population, with distinct gene signatures and functions in liver fibrosis and liver cancer (299). Further research will help to gain a better understanding of the different hepatic macrophage subtypes in mice and humans, and a better definition of their disease-promoting phenotype and the key disease-related ‘macrophage switches’ will allow the development of new macrophage-targeted therapies.

Like macrophages, platelets can also have opposing functions in patients with chronic liver disease since both low and high platelet counts have been related to a poor prognosis in patients with HCC (300). Several studies using rodent models of chronic liver diseases and HCC demonstrated that antiplatelet therapy, e.g., aspirin and clopidogrel, can ameliorate liver injury and disease outcomes. However, future research will be important to better clarify the functional role of platelets in liver disease in relation to disease stage, such as early vs late stages, and acute vs chronic disease. Until now, there is no recommendation for the use of antiplatelet therapy in patients with liver disease since its use requires careful monitoring due to possible bleeding complications, especially in patients with cirrhosis. Here, further studies with more specific antiplatelet agents, like GPVI, PAR4, or PI3K inhibitors may reduce possible bleeding complications (301). Platelets can influence macrophage differentiation and polarization through direct cell-cell interaction and the release of growth factors, cytokines, chemokines, and other mediators, affecting their pro-inflammatory, anti-tumor, and profibrotic phenotype, offering platelet-targeted treatment approaches (288). Finally, platelets may also be used as a therapeutic delivery system, supporting optimized tumor therapy (302). Such platelet-targeted strategy, especially when combined with a macrophage-targeted approach, could reduce adverse effects and enhance therapeutic efficacy in liver fibrosis and cancer (303). In conclusion, shedding light on the interplay between macrophages and platelets, and possibly other immune cells involved, may open new avenues to develop effective therapies for liver fibrosis and HCC.

## Author contributions

MC: Writing – original draft, Writing – review & editing. DoS: Writing – original draft, Writing – review & editing. CD: Conceptualization, Writing – original draft, Writing – review & editing. DeS: Conceptualization, Writing – original draft, Writing – review & editing.
